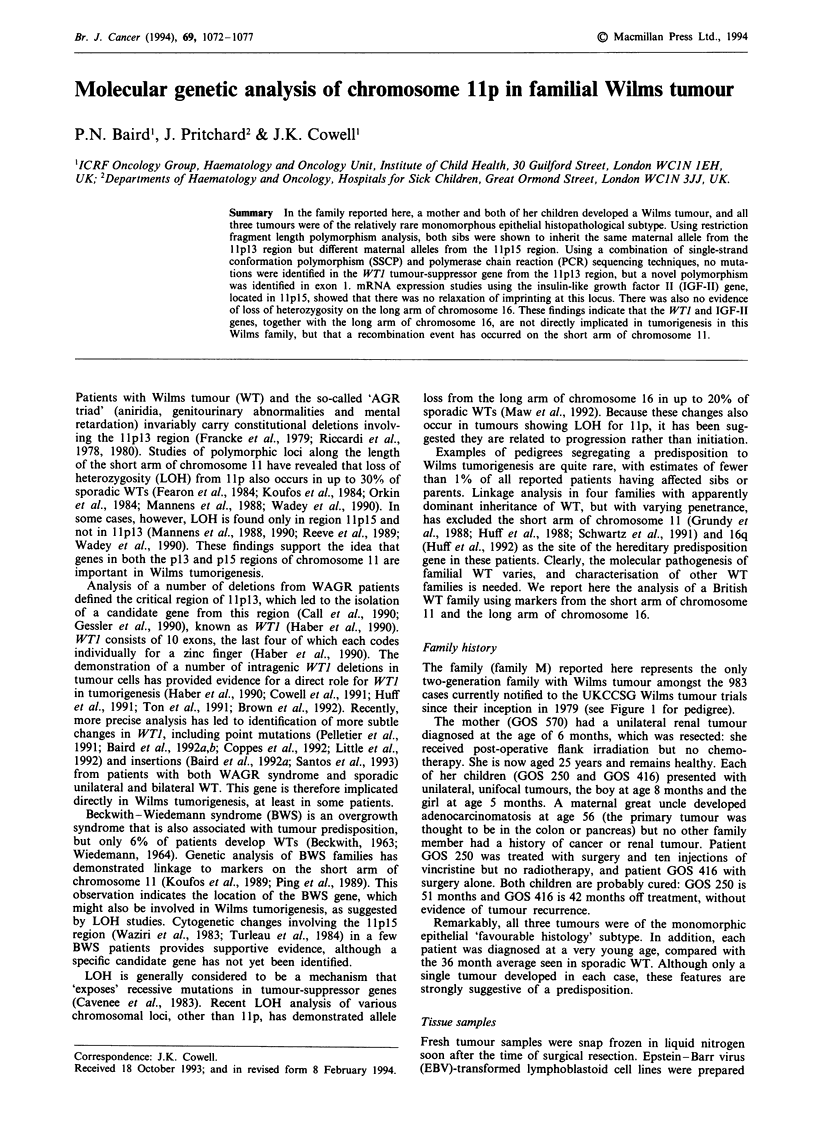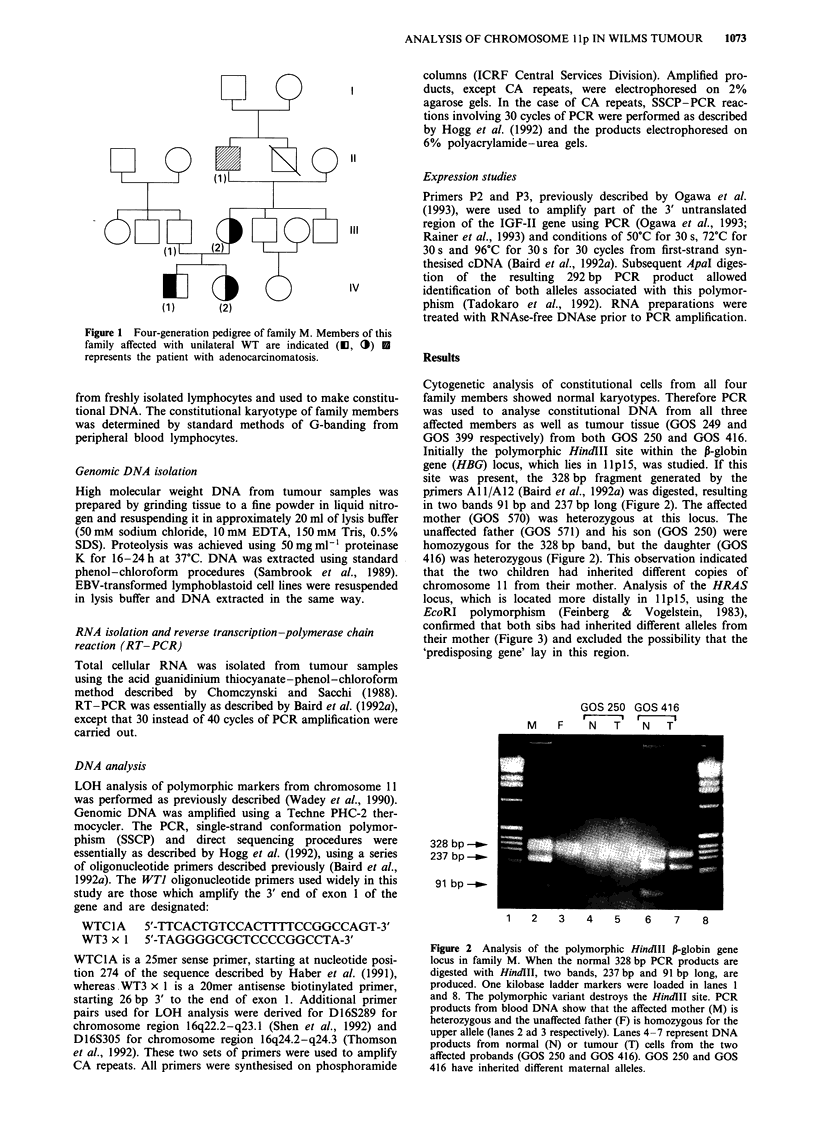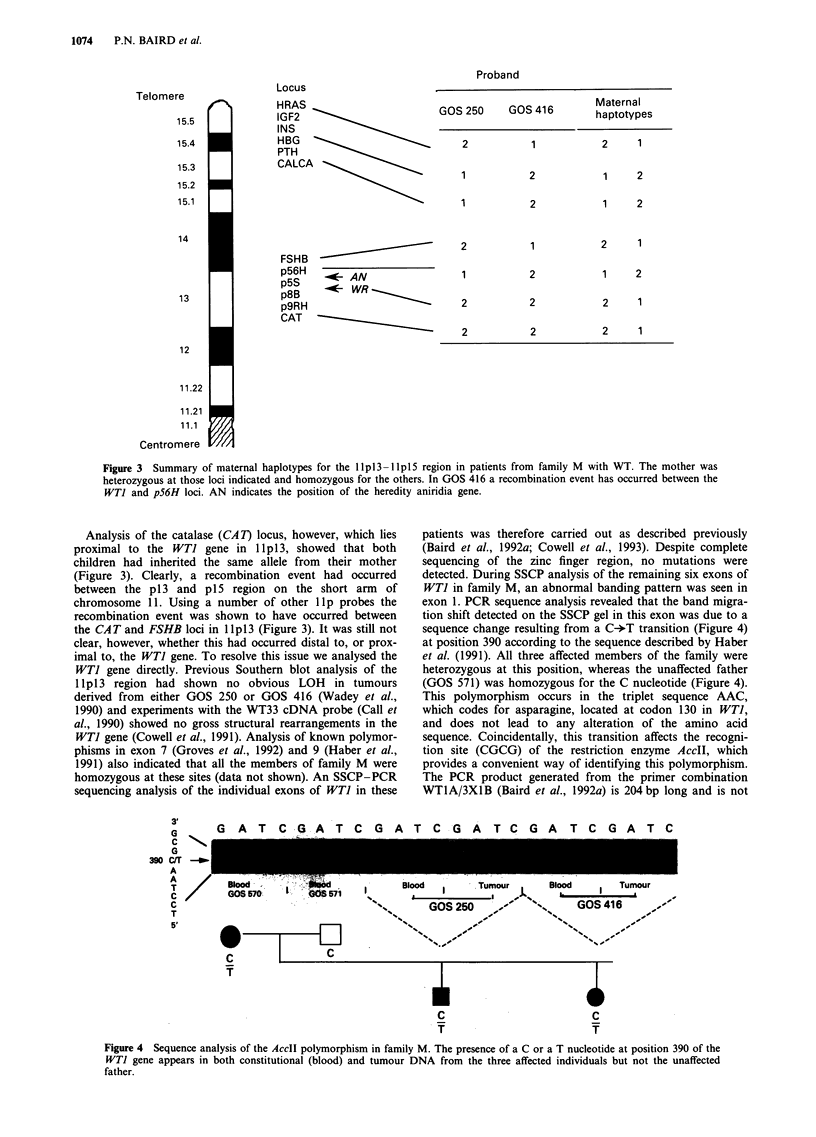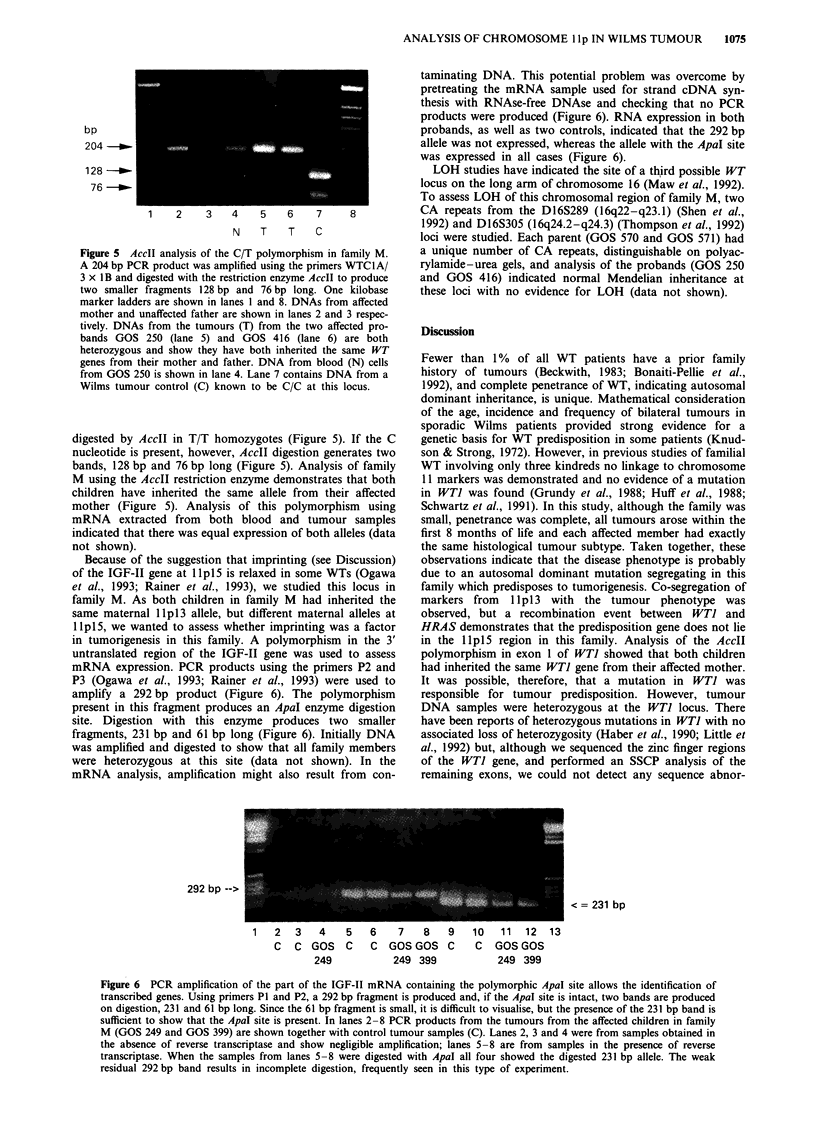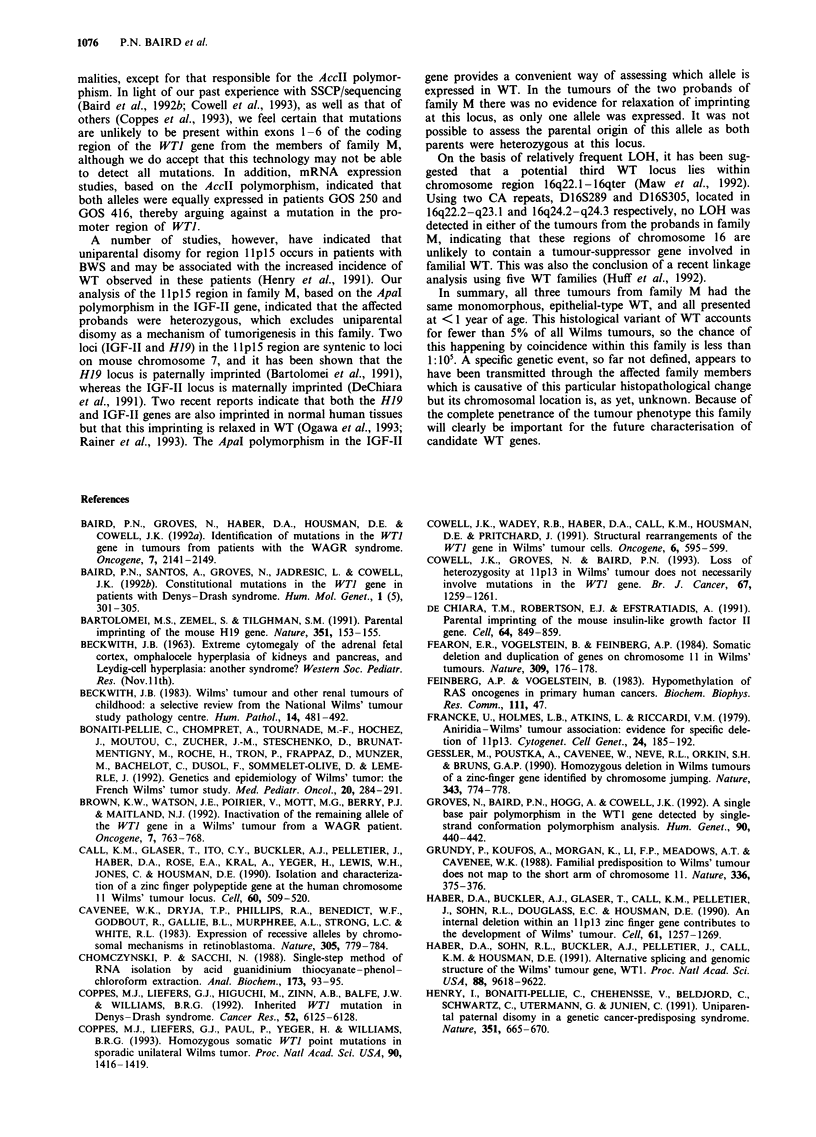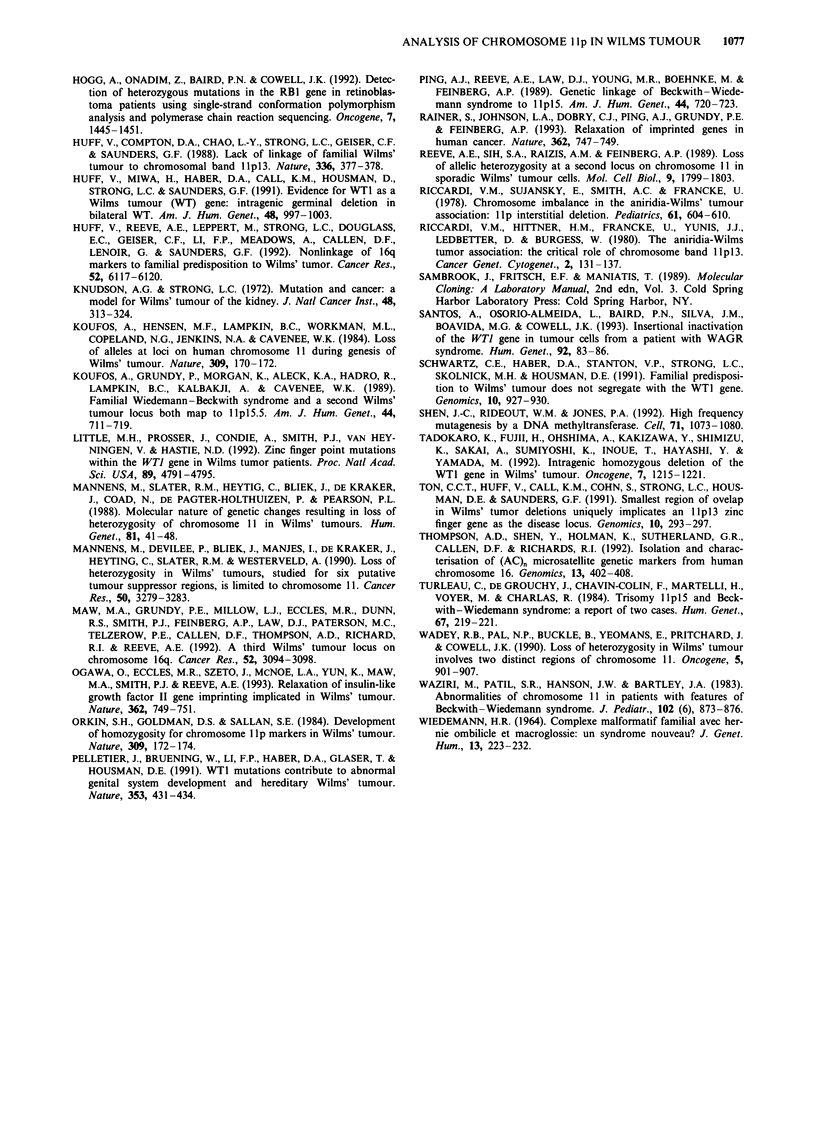# Molecular genetic analysis of chromosome 11p in familial Wilms tumour.

**DOI:** 10.1038/bjc.1994.210

**Published:** 1994-06

**Authors:** P. N. Baird, J. Pritchard, J. K. Cowell

**Affiliations:** Haematology and Oncology Unit, Institute of Child Health, London, UK.

## Abstract

**Images:**


Br. J. Cancer (1994), 69, 1072–1077 © Macmillan Press Ltd., 1994

### Molecular genetic analysis of chromosome 11p in familial Wilms tumour

P.N. Baird[1], J. Pritchard[2] & J.K. Cowell[1]

[1]ICRF Oncology Group, Haematology and Oncology Unit, Institute of Child Health, 30 Guilford Street, London WC1N 1EH, UK; [2]Departments of Haematology and Oncology, Hospitals for Sick Children, Great Ormond Street, London WC1N 3JJ, UK.

**Summary** In the family reported here, a mother and both of her children developed a Wilms tumour, and all three tumours were of the relatively rare monomorphous epithelial histopathological subtype. Using restriction fragment length polymorphism analysis, both sibs were shown to inherit the same maternal allele from the 11p13 region but different maternal alleles from the 11p15 region. Using a combination of single-strand conformation polymorphism (SSCP) and polymerase chain reaction (PCR) sequencing techniques, no mutations were identified in the *WT1* tumour-suppressor gene from the 11p13 region, but a novel polymorphism was identified in exon 1. mRNA expression studies using the insulin-like growth factor II (IGF-II) gene, located in 11p15, showed that there was no relaxation of imprinting at this locus. There was also no evidence of loss of heterozygosity on the long arm of chromosome 16. These findings indicate that the *WT1* and IGF-II genes, together with the long arm of chromosome 16, are not directly implicated in tumorigenesis in this Wilms family, but that a recombination event has occurred on the short arm of chromosome 11.

Patients with Wilms tumour (WT) and the so-called 'AGR triad' (aniridia, genitourinary abnormalities and mental retardation) invariably carry constitutional deletions involving the 11p13 region (Francke *et al.*, 1979; Riccardi *et al.*, 1978, 1980). Studies of polymorphic loci along the length of the short arm of chromosome 11 have revealed that loss of heterozygosity (LOH) from 11p also occurs in up to 30% of sporadic WTs (Fearon *et al.*, 1984; Koufos *et al.*, 1984; Orkin *et al.*, 1984; Mannens *et al.*, 1988; Wadey *et al.*, 1990). In some cases, however, LOH is found only in region 11p15 and not in 11p13 (Mannens *et al.*, 1988, 1990; Reeve *et al.*, 1989; Wadey *et al.*, 1990). These findings support the idea that genes in both the p13 and p15 regions of chromosome 11 are important in Wilms tumorigenesis.

Analysis of a number of deletions from WAGR patients defined the critical region of 11p13, which led to the isolation of a candidate gene from this region (Call *et al.*, 1990; Gessler *et al.*, 1990), known as *WT1* (Haber *et al.*, 1990). *WT1* consists of 10 exons, the last four of which each codes individually for a zinc finger (Haber *et al.*, 1990). The demonstration of a number of intragenic *WT1* deletions in tumour cells has provided evidence for a direct role for *WT1* in tumorigenesis (Haber *et al.*, 1990; Cowell *et al.*, 1991; Huff *et al.*, 1991; Ton *et al.*, 1991; Brown *et al.*, 1992). Recently, more precise analysis has led to identification of more subtle changes in *WT1*, including point mutations (Pelletier *et al.*, 1991; Baird *et al.*, 1992*a*,*b*; Coppes *et al.*, 1992; Little *et al.*, 1992) and insertions (Baird *et al.*, 1992*a*; Santos *et al.*, 1993) from patients with both WAGR syndrome and sporadic unilateral and bilateral WT. This gene is therefore implicated directly in Wilms tumorigenesis, at least in some patients.

Beckwith–Wiedemann syndrome (BWS) is an overgrowth syndrome that is also associated with tumour predisposition, but only 6% of patients develop WTs (Beckwith, 1963; Wiedemann, 1964). Genetic analysis of BWS families has demonstrated linkage to markers on the short arm of chromosome 11 (Koufos *et al.*, 1989; Ping *et al.*, 1989). This observation indicates the location of the BWS gene, which might also be involved in Wilms tumorigenesis, as suggested by LOH studies. Cytogenetic changes involving the 11p15 region (Waziri *et al.*, 1983; Turleau *et al.*, 1984) in a few BWS patients provides supportive evidence, although a specific candidate gene has not yet been identified.

LOH is generally considered to be a mechanism that 'exposes' recessive mutations in tumour-suppressor genes (Cavenee *et al.*, 1983). Recent LOH analysis of various chromosomal loci, other than 11p, has demonstrated allele loss from the long arm of chromosome 16 in up to 20% of sporadic WTs (Maw *et al.*, 1992). Because these changes also occur in tumours showing LOH for 11p, it has been suggested they are related to progression rather than initiation.

Examples of pedigrees segregating a predisposition to Wilms tumorigenesis are quite rare, with estimates of fewer than 1% of all reported patients having affected sibs or parents. Linkage analysis in four families with apparently dominant inheritance of WT, but with varying penetrance, has excluded the short arm of chromosome 11 (Grundy *et al.*, 1988; Huff *et al.*, 1988; Schwartz *et al.*, 1991) and 16q (Huff *et al.*, 1992) as the site of the hereditary predisposition gene in these patients. Clearly, the molecular pathogenesis of familial WT varies, and characterisation of other WT families is needed. We report here the analysis of a British WT family using markers from the short arm of chromosome 11 and the long arm of chromosome 16.

### *Family history*

The family (family M) reported here represents the only two-generation family with Wilms tumour amongst the 983 cases currently notified to the UKCCSG Wilms tumour trials since their inception in 1979 (see Figure 1 for pedigree).

The mother (GOS 570) had a unilateral renal tumour diagnosed at the age of 6 months, which was resected: she received post-operative flank irradiation but no chemotherapy. She is now aged 25 years and remains healthy. Each of her children (GOS 250 and GOS 416) presented with unilateral, unifocal tumours, the boy at age 8 months and the girl at age 5 months. A maternal great uncle developed adenocarcinomatosis at age 56 (the primary tumour was thought to be in the colon or pancreas) but no other family member had a history of cancer or renal tumour. Patient GOS 250 was treated with surgery and ten injections of vincristine but no radiotherapy, and patient GOS 416 with surgery alone. Both children are probably cured: GOS 250 is 51 months and GOS 416 is 42 months off treatment, without evidence of tumour recurrence.

Remarkably, all three tumours were of the monomorphic epithelial 'favourable histology' subtype. In addition, each patient was diagnosed at a very young age, compared with the 36 month average seen in sporadic WT. Although only a single tumour developed in each case, these features are strongly suggestive of a predisposition.

### *Tissue samples*

Fresh tumour samples were snap frozen in liquid nitrogen soon after the time of surgical resection. Epstein–Barr virus (EBV)-transformed lymphoblastoid cell lines were prepared

Correspondence: J.K. Cowell.
Received 18 October 1993; and in revised form 8 February 1994.

**Figure 1** Four-generation pedigree of family M. Members of this family affected with unilateral WT are indicated (◧, ◑) ▨ represents the patient with adenocarcinomatosis.

from freshly isolated lymphocytes and used to make constitutional DNA. The constitutional karyotype of family members was determined by standard methods of G-banding from peripheral blood lymphocytes.

*Genomic DNA isolation*

High molecular weight DNA from tumour samples was prepared by grinding tissue to a fine powder in liquid nitrogen and resuspending it in approximately 20 ml of lysis buffer (50 mM sodium chloride, 10 mM EDTA, 150 mM Tris, 0.5% SDS). Proteolysis was achieved using 50 mg ml$^{-1}$ proteinase K for 16–24 h at 37°C. DNA was extracted using standard phenol–chloroform procedures (Sambrook *et al.*, 1989). EBV-transformed lymphoblastoid cell lines were resuspended in lysis buffer and DNA extracted in the same way.

*RNA isolation and reverse transcription–polymerase chain reaction (RT–PCR)*

Total cellular RNA was isolated from tumour samples using the acid guanidinium thiocyanate–phenol–chloroform method described by Chomczynski and Sacchi (1988). RT–PCR was essentially as described by Baird *et al.* (1992*a*), except that 30 instead of 40 cycles of PCR amplification were carried out.

*DNA analysis*

LOH analysis of polymorphic markers from chromosome 11 was performed as previously described (Wadey *et al.*, 1990). Genomic DNA was amplified using a Techne PHC-2 thermocycler. The PCR, single-strand conformation polymorphism (SSCP) and direct sequencing procedures were essentially as described by Hogg *et al.* (1992), using a series of oligonucleotide primers described previously (Baird *et al.*, 1992*a*). The *WT1* oligonucleotide primers used widely in this study are those which amplify the 3′ end of exon 1 of the gene and are designated:

WTC1A 5′-TTCACTGTCCACTTTTCCGGCCAGT-3′

WT3 × 1 5′-TAGGGGCGCTCCCCGGCCTA-3′

WTC1A is a 25mer sense primer, starting at nucleotide position 274 of the sequence described by Haber *et al.* (1991), whereas WT3 × 1 is a 20mer antisense biotinylated primer, starting 26 bp 3′ to the end of exon 1. Additional primer pairs used for LOH analysis were derived for D16S289 for chromosome region 16q22.2–q23.1 (Shen *et al.*, 1992) and D16S305 for chromosome region 16q24.2–q24.3 (Thomson *et al.*, 1992). These two sets of primers were used to amplify CA repeats. All primers were synthesised on phosphoramide columns (ICRF Central Services Division). Amplified products, except CA repeats, were electrophoresed on 2% agarose gels. In the case of CA repeats, SSCP–PCR reactions involving 30 cycles of PCR were performed as described by Hogg *et al.* (1992) and the products electrophoresed on 6% polyacrylamide–urea gels.

*Expression studies*

Primers P2 and P3, previously described by Ogawa *et al.* (1993), were used to amplify part of the 3′ untranslated region of the IGF-II gene using PCR (Ogawa *et al.*, 1993; Rainer *et al.*, 1993) and conditions of 50°C for 30 s, 72°C for 30 s and 96°C for 30 s for 30 cycles from first-strand synthesised cDNA (Baird *et al.*, 1992*a*). Subsequent *Apa*I digestion of the resulting 292 bp PCR product allowed identification of both alleles associated with this polymorphism (Tadokaro *et al.*, 1992). RNA preparations were treated with RNAse-free DNAse prior to PCR amplification.

### Results

Cytogenetic analysis of constitutional cells from all four family members showed normal karyotypes. Therefore PCR was used to analyse constitutional DNA from all three affected members as well as tumour tissue (GOS 249 and GOS 399 respectively) from both GOS 250 and GOS 416. Initially the polymorphic *Hin*dIII site within the β-globin gene (*HBG*) locus, which lies in 11p15, was studied. If this site was present, the 328 bp fragment generated by the primers A11/A12 (Baird *et al.*, 1992*a*) was digested, resulting in two bands 91 bp and 237 bp long (Figure 2). The affected mother (GOS 570) was heterozygous at this locus. The unaffected father (GOS 571) and his son (GOS 250) were homozygous for the 328 bp band, but the daughter (GOS 416) was heterozygous (Figure 2). This observation indicated that the two children had inherited different copies of chromosome 11 from their mother. Analysis of the *HRAS* locus, which is located more distally in 11p15, using the *Eco*RI polymorphism (Feinberg & Vogelstein, 1983), confirmed that both sibs had inherited different alleles from their mother (Figure 3) and excluded the possibility that the 'predisposing gene' lay in this region.

**Figure 2** Analysis of the polymorphic *Hin*dIII β-globin gene locus in family M. When the normal 328 bp PCR products are digested with *Hin*dIII, two bands, 237 bp and 91 bp long, are produced. One kilobase ladder markers were loaded in lanes 1 and 8. The polymorphic variant destroys the *Hin*dIII site. PCR products from blood DNA show that the affected mother (M) is heterozygous and the unaffected father (F) is homozygous for the upper allele (lanes 2 ad 3 respectively). Lanes 4–7 represent DNA products from normal (N) or tumour (T) cells from the two affected probands (GOS 250 and GOS 416). GOS 250 and GOS 416 have inherited different maternal alleles.

**Figure 3** Summary of maternal haplotypes for the 11p13–11p15 region in patients from family M with WT. The mother was heterozygous at those loci indicated and homozygous for the others. In GOS 416 a recombination event has occurred between the *WT1* and *p56H* loci. AN indicates the position of the heredity aniridia gene.

Analysis of the catalase (*CAT*) locus, however, which lies proximal to the *WT1* gene in 11p13, showed that both children had inherited the same allele from their mother (Figure 3). Clearly, a recombination event had occurred between the p13 and p15 region on the short arm of chromosome 11. Using a number of other 11p probes the recombination event was shown to have occurred between the *CAT* and *FSHB* loci in 11p13 (Figure 3). It was still not clear, however, whether this had occurred distal to, or proximal to, the *WT1* gene. To resolve this issue we analysed the *WT1* gene directly. Previous Southern blot analysis of the 11p13 region had shown no obvious LOH in tumours derived from either GOS 250 or GOS 416 (Wadey *et al.*, 1990) and experiments with the WT33 cDNA probe (Call *et al.*, 1990) showed no gross structural rearrangements in the *WT1* gene (Cowell *et al.*, 1991). Analysis of known polymorphisms in exon 7 (Groves *et al.*, 1992) and 9 (Haber *et al.*, 1991) also indicated that all the members of family M were homozygous at these sites (data not shown). An SSCP-PCR sequencing analysis of the individual exons of *WT1* in these patients was therefore carried out as described previously (Baird *et al.*, 1992*a*; Cowell *et al.*, 1993). Despite complete sequencing of the zinc finger region, no mutations were detected. During SSCP analysis of the remaining six exons of *WT1* in family M, an abnormal banding pattern was seen in exon 1. PCR sequence analysis revealed that the band migration shift detected on the SSCP gel in this exon was due to a sequence change resulting from a C→T transition (Figure 4) at position 390 according to the sequence described by Haber *et al.* (1991). All three affected members of the family were heterozygous at this position, whereas the unaffected father (GOS 571) was homozygous for the C nucleotide (Figure 4). This polymorphism occurs in the triplet sequence AAC, which codes for asparagine, located at codon 130 in *WT1*, and does not lead to any alteration of the amino acid sequence. Coincidentally, this transition affects the recognition site (CGCG) of the restriction enzyme *Acc*II, which provides a convenient way of identifying this polymorphism. The PCR product generated from the primer combination WT1A/3X1B (Baird *et al.*, 1992*a*) is 204 bp long and is not

**Figure 4** Sequence analysis of the *Acc*II polymorphism in family M. The presence of a C or a T nucleotide at position 390 of the *WT1* gene appears in both constitutional (blood) and tumour DNA from the three affected individuals but not the unaffected father.

**Figure 5** *Acc*II analysis of the C/T polymorphism in family M. A 204 bp PCR product was amplified using the primers WTC1A/3 × 1B and digested with the restriction enzyme *Acc*II to produce two smaller fragments 128 bp and 76 bp long. One kilobase marker ladders are shown in lanes 1 and 8. DNAs from affected mother and unaffected father are shown in lanes 2 and 3 respectively. DNAs from the tumours (T) from the two affected probands GOS 250 (lane 5) and GOS 416 (lane 6) are both heterozygous and show they have both inherited the same *WT* genes from their mother and father. DNA from blood (N) cells from GOS 250 is shown in lane 4. Lane 7 contains DNA from a Wilms tumour control (C) known to be C/C at this locus.

digested by *Acc*II in T/T homozygotes (Figure 5). If the C nucleotide is present, however, *Acc*II digestion generates two bands, 128 bp and 76 bp long (Figure 5). Analysis of family M using the *Acc*II restriction enzyme demonstrates that both children have inherited the same allele from their affected mother (Figure 5). Analysis of this polymorphism using mRNA extracted from both blood and tumour samples indicated that there was equal expression of both alleles (data not shown).

Because of the suggestion that imprinting (see Discussion) of the IGF-II gene at 11p15 is relaxed in some WTs (Ogawa *et al.*, 1993; Rainer *et al.*, 1993), we studied this locus in family M. As both children in family M had inherited the same maternal 11p13 allele, but different maternal alleles at 11p15, we wanted to assess whether imprinting was a factor in tumorigenesis in this family. A polymorphism in the 3′ untranslated region of the IGF-II gene was used to assess mRNA expression. PCR products using the primers P2 and P3 (Ogawa *et al.*, 1993; Rainer *et al.*, 1993) were used to amplify a 292 bp product (Figure 6). The polymorphism present in this fragment produces an *Apa*I enzyme digestion site. Digestion with this enzyme produces two smaller fragments, 231 bp and 61 bp long (Figure 6). Initially DNA was amplified and digested to show that all family members were heterozygous at this site (data not shown). In the mRNA analysis, amplification might also result from contaminating DNA. This potential problem was overcome by pretreating the mRNA sample used for strand cDNA synthesis with RNAse-free DNAse and checking that no PCR products were produced (Figure 6). RNA expression in both probands, as well as two controls, indicated that the 292 bp allele was not expressed, whereas the allele with the *Apa*I site was expressed in all cases (Figure 6).

LOH studies have indicated the site of a third possible *WT* locus on the long arm of chromosome 16 (Maw *et al.*, 1992). To assess LOH of this chromosomal region of family M, two CA repeats from the D16S289 (16q22–q23.1) (Shen *et al.*, 1992) and D16S305 (16q24.2–q24.3) (Thompson *et al.*, 1992) loci were studied. Each parent (GOS 570 and GOS 571) had a unique number of CA repeats, distinguishable on polyacrylamide–urea gels, and analysis of the probands (GOS 250 and GOS 416) indicated normal Mendelian inheritance at these loci with no evidence for LOH (data not shown).

### Discussion

Fewer than 1% of all WT patients have a prior family history of tumours (Beckwith, 1983; Bonaiti-Pellie *et al.*, 1992), and complete penetrance of WT, indicating autosomal dominant inheritance, is unique. Mathematical consideration of the age, incidence and frequency of bilateral tumours in sporadic Wilms patients provided strong evidence for a genetic basis for WT predisposition in some patients (Knudson & Strong, 1972). However, in previous studies of familial WT involving only three kindreds no linkage to chromosome 11 markers was demonstrated and no evidence of a mutation in *WT1* was found (Grundy *et al.*, 1988; Huff *et al.*, 1988; Schwartz *et al.*, 1991). In this study, although the family was small, penetrance was complete, all tumours arose within the first 8 months of life and each affected member had exactly the same histological tumour subtype. Taken together, these observations indicate that the disease phenotype is probably due to an autosomal dominant mutation segregating in this family which predisposes to tumorigenesis. Co-segregation of markers from 11p13 with the tumour phenotype was observed, but a recombination event between *WT1* and *HRAS* demonstrates that the predisposition gene does not lie in the 11p15 region in this family. Analysis of the *Acc*II polymorphism in exon 1 of *WT1* showed that both children had inherited the same *WT1* gene from their affected mother. It was possible, therefore, that a mutation in *WT1* was responsible for tumour predisposition. However, tumour DNA samples were heterozygous at the *WT1* locus. There have been reports of heterozygous mutations in *WT1* with no associated loss of heterozygosity (Haber *et al.*, 1990; Little *et al.*, 1992) but, although we sequenced the zinc finger regions of the *WT1* gene, and performed an SSCP analysis of the remaining exons, we could not detect any sequence abnor-

**Figure 6** PCR amplification of the part of the IGF-II mRNA containing the polymorphic *Apa*I site allows the identification of transcribed genes. Using primers P1 and P2, a 292 bp fragment is produced and, if the *Apa*I site is intact, two bands are produced on digestion, 231 and 61 bp long. Since the 61 bp fragment is small, it is difficult to visualise, but the presence of the 231 bp band is sufficient to show that the *Apa*I site is present. In lanes 2–8 PCR products from the tumours from the affected children in family M (GOS 249 and GOS 399) are shown together with control tumour samples (C). Lanes 2, 3 and 4 were from samples obtained in the absence of reverse transcriptase and show negligible amplification; lanes 5–8 are from samples in the presence of reverse transcriptase. When the samples from lanes 5–8 were digested with *Apa*I all four showed the digested 231 bp allele. The weak residual 292 bp band results in incomplete digestion, frequently seen in this type of experiment.

malities, except for that responsible for the *Acc*II polymorphism. In light of our past experience with SSCP/sequencing (Baird et al., 1992*b*; Cowell et al., 1993), as well as that of others (Coppes et al., 1993), we feel certain that mutations are unlikely to be present within exons 1–6 of the coding region of the *WT1* gene from the members of family M, although we do accept that this technology may not be able to detect all mutations. In addition, mRNA expression studies, based on the *Acc*II polymorphism, indicated that both alleles were equally expressed in patients GOS 250 and GOS 416, thereby arguing against a mutation in the promoter region of *WT1*.

A number of studies, however, have indicated that uniparental disomy for region 11p15 occurs in patients with BWS and may be associated with the increased incidence of WT observed in these patients (Henry et al., 1991). Our analysis of the 11p15 region in family M, based on the *Apa*I polymorphism in the IGF-II gene, indicated that the affected probands were heterozygous, which excludes uniparental disomy as a mechanism of tumorigenesis in this family. Two loci (IGF-II and *H19*) in the 11p15 region are syntenic to loci on mouse chromosome 7, and it has been shown that the *H19* locus is paternally imprinted (Bartolomei et al., 1991), whereas the IGF-II locus is maternally imprinted (DeChiara et al., 1991). Two recent reports indicate that both the *H19* and IGF-II genes are also imprinted in normal human tissues but that this imprinting is relaxed in WT (Ogawa et al., 1993; Rainer et al., 1993). The *Apa*I polymorphism in the IGF-II gene provides a convenient way of assessing which allele is expressed in WT. In the tumours of the two probands of family M there was no evidence for relaxation of imprinting at this locus, as only one allele was expressed. It was not possible to assess the parental origin of this allele as both parents were heterozygous at this locus.

On the basis of relatively frequent LOH, it has been suggested that a potential third WT locus lies within chromosome region 16q22.1–16qter (Maw et al., 1992). Using two CA repeats, D16S289 and D16S305, located in 16q22.2–q23.1 and 16q24.2–q24.3 respectively, no LOH was detected in either of the tumours from the probands in family M, indicating that these regions of chromosome 16 are unlikely to contain a tumour-suppressor gene involved in familial WT. This was also the conclusion of a recent linkage analysis using five WT families (Huff et al., 1992).

In summary, all three tumours from family M had the same monomorphous, epithelial-type WT, and all presented at $<1$ year of age. This histological variant of WT accounts for fewer than 5% of all Wilms tumours, so the chance of this happening by coincidence within this family is less than $1:10^5$. A specific genetic event, so far not defined, appears to have been transmitted through the affected family members which is causative of this particular histopathological change but its chromosomal location is, as yet, unknown. Because of the complete penetrance of the tumour phenotype this family will clearly be important for the future characterisation of candidate WT genes.

### References

BAIRD, P.N., GROVES, N., HABER, D.A., HOUSMAN, D.E. & COWELL, J.K. (1992*a*). Identification of mutations in the *WT1* gene in tumours from patients with the WAGR syndrome. *Oncogene*, **7**, 2141–2149.

BAIRD, P.N., SANTOS, A., GROVES, N., JADRESIC, L. & COWELL, J.K. (1992*b*). Constitutional mutations in the *WT1* gene in patients with Denys–Drash syndrome. *Hum. Mol. Genet.*, **1** (5), 301–305.

BARTOLOMEI, M.S., ZEMEL, S. & TILGHMAN, S.M. (1991). Parental imprinting of the mouse H19 gene. *Nature*, **351**, 153–155.

BECKWITH, J.B. (1963). Extreme cytomegaly of the adrenal fetal cortex, omphalocele hyperplasia of kidneys and pancreas, and Leydig-cell hyperplasia: another syndrome? *Western Soc. Pediatr. Res.* (Nov.11th).

BECKWITH, J.B. (1983). Wilms' tumour and other renal tumours of childhood: a selective review from the National Wilms' tumour study pathology centre. *Hum. Pathol.*, **14**, 481–492.

BONAITI-PELLIE, C., CHOMPRET, A., TOURNADE, M.-F., HOCHEZ, J., MOUTOU, C., ZUCHER, J.-M., STESCHENKO, D., BRUNAT-MENTIGNY, M., ROCHE, H., TRON, P., FRAPPAZ, D., MUNZER, M., BACHELOT, C., DUSOL, F., SOMMELET-OLIVE, D. & LEMERLE, J. (1992). Genetics and epidemiology of Wilms' tumor: the French Wilms' tumor study. *Med. Pediatr. Oncol.*, **20**, 284–291.

BROWN, K.W., WATSON, J.E., POIRIER, V., MOTT, M.G., BERRY, P.J. & MAITLAND, N.J. (1992). Inactivation of the remaining allele of the *WT1* gene in a Wilms' tumour from a WAGR patient. *Oncogene*, **7**, 763–768.

CALL, K.M., GLASER, T., ITO, C.Y., BUCKLER, A.J., PELLETIER, J., HABER, D.A., ROSE, E.A., KRAL, A., YEGER, H., LEWIS, W.H., JONES, C. & HOUSMAN, D.E. (1990). Isolation and characterization of a zinc finger polypeptide gene at the human chromosome 11 Wilms' tumour locus. *Cell*, **60**, 509–520.

CAVENEE, W.K., DRYJA, T.P., PHILLIPS, R.A., BENEDICT, W.F., GODBOUT, R., GALLIE, B.L., MURPHREE, A.L., STRONG, L.C. & WHITE, R.L. (1983). Expression of recessive alleles by chromosomal mechanisms in retinoblastoma. *Nature*, **305**, 779–784.

CHOMCZYNSKI, P. & SACCHI, N. (1988). Single-step method of RNA isolation by acid guanidinium thiocyanate-phenol-chloroform extraction. *Anal. Biochem.*, **173**, 93–95.

COPPES, M.J., LIEFERS, G.J., HIGUCHI, M., ZINN, A.B., BALFE, J.W. & WILLIAMS, B.R.G. (1992). Inherited *WT1* mutation in Denys–Drash syndrome. *Cancer Res.*, **52**, 6125–6128.

COPPES, M.J., LIEFERS, G.J., PAUL, P., YEGER, H. & WILLIAMS, B.R.G. (1993). Homozygous somatic *WT1* point mutations in sporadic unilateral Wilms tumor. *Proc. Natl Acad. Sci. USA*, **90**, 1416–1419.

COWELL, J.K., WADEY, R.B., HABER, D.A., CALL, K.M., HOUSMAN, D.E. & PRITCHARD, J. (1991). Structural rearrangements of the *WT1* gene in Wilms' tumour cells. *Oncogene*, **6**, 595–599.

COWELL, J.K., GROVES, N. & BAIRD, P.N. (1993). Loss of heterozygosity at 11p13 in Wilms' tumour does not necessarily involve mutations in the *WT1* gene. *Br. J. Cancer*, **67**, 1259–1261.

DE CHIARA, T.M., ROBERTSON, E.J. & EFSTRATIADIS, A. (1991). Parental imprinting of the mouse insulin-like growth factor II gene. *Cell*, **64**, 849–859.

FEARON, E.R., VOGELSTEIN, B. & FEINBERG, A.P. (1984). Somatic deletion and duplication of genes on chromosome 11 in Wilms' tumours. *Nature*, **309**, 176–178.

FEINBERG, A.P. & VOGELSTEIN, B. (1983). Hypomethylation of RAS oncogenes in primary human cancers. *Biochem. Biophys. Res. Comm.*, **111**, 47.

FRANCKE, U., HOLMES, L.B., ATKINS, L. & RICCARDI, V.M. (1979). Aniridia–Wilms' tumour association: evidence for specific deletion of 11p13. *Cytogenet. Cell Genet.*, **24**, 185–192.

GESSLER, M., POUSTKA, A., CAVENEE, W., NEVE, R.L., ORKIN, S.H. & BRUNS, G.A.P. (1990). Homozygous deletion in Wilms tumours of a zinc-finger gene identified by chromosome jumping. *Nature*, **343**, 774–778.

GROVES, N., BAIRD, P.N., HOGG, A. & COWELL, J.K. (1992). A single base pair polymorphism in the WT1 gene detected by single-strand conformation polymorphism analysis. *Hum. Genet.*, **90**, 440–442.

GRUNDY, P., KOUFOS, A., MORGAN, K., LI, F.P., MEADOWS, A.T. & CAVENEE, W.K. (1988). Familial predisposition to Wilms' tumour does not map to the short arm of chromosome 11. *Nature*, **336**, 375–376.

HABER, D.A., BUCKLER, A.J., GLASER, T., CALL, K.M., PELLETIER, J., SOHN, R.L., DOUGLASS, E.C. & HOUSMAN, D.E. (1990). An internal deletion within an 11p13 zinc finger gene contributes to the development of Wilms' tumour. *Cell*, **61**, 1257–1269.

HABER, D.A., SOHN, R.L., BUCKLER, A.J., PELLETIER, J., CALL, K.M. & HOUSMAN, D.E. (1991). Alternative splicing and genomic structure of the Wilms' tumour gene, WT1. *Proc. Natl Acad. Sci. USA*, **88**, 9618–9622.

HENRY, I., BONAITI-PELLIE, C., CHEHENSSE, V., BELDJORD, C., SCHWARTZ, C., UTERMANN, G. & JUNIEN, C. (1991). Uniparental paternal disomy in a genetic cancer-predisposing syndrome. *Nature*, **351**, 665–670.

HOGG, A., ONADIM, Z., BAIRD, P.N. & COWELL, J.K. (1992). Detection of heterozygous mutations in the RB1 gene in retinoblastoma patients using single-strand conformation polymorphism analysis and polymerase chain reaction sequencing. *Oncogene*, **7**, 1445–1451.

HUFF, V., COMPTON, D.A., CHAO, L.-Y., STRONG, L.C., GEISER, C.F. & SAUNDERS, G.F. (1988). Lack of linkage of familial Wilms' tumour to chromosomal band 11p13. *Nature*, **336**, 377–378.

HUFF, V., MIWA, H., HABER, D.A., CALL, K.M., HOUSMAN, D., STRONG, L.C. & SAUNDERS, G.F. (1991). Evidence for WT1 as a Wilms tumour (WT) gene: intragenic germinal deletion in bilateral WT. *Am. J. Hum. Genet.*, **48**, 997–1003.

HUFF, V., REEVE, A.E., LEPPERT, M., STRONG, L.C., DOUGLASS, E.C., GEISER, C.F., LI, F.P., MEADOWS, A., CALLEN, D.F., LENOIR, G. & SAUNDERS, G.F. (1992). Nonlinkage of 16q markers to familial predisposition to Wilms' tumor. *Cancer Res.*, **52**, 6117–6120.

KNUDSON, A.G. & STRONG, L.C. (1972). Mutation and cancer: a model for Wilms' tumour of the kidney. *J. Natl Cancer Inst.*, **48**, 313–324.

KOUFOS, A., HENSEN, M.F., LAMPKIN, B.C., WORKMAN, M.L., COPELAND, N.G., JENKINS, N.A. & CAVENEE, W.K. (1984). Loss of alleles at loci on human chromosome 11 during genesis of Wilms' tumour. *Nature*, **309**, 170–172.

KOUFOS, A., GRUNDY, P., MORGAN, K., ALECK, K.A., HADRO, R., LAMPKIN, B.C., KALBAKJI, A. & CAVENEE, W.K. (1989). Familial Wiedemann–Beckwith syndrome and a second Wilms' tumour locus both map to 11p15.5. *Am. J. Hum. Genet.*, **44**, 711–719.

LITTLE, M.H., PROSSER, J., CONDIE, A., SMITH, P.J., VAN HEYNINGEN, V. & HASTIE, N.D. (1992). Zinc finger point mutations within the *WT1* gene in Wilms tumor patients. *Proc. Natl Acad. Sci. USA*, **89**, 4791–4795.

MANNENS, M., SLATER, R.M., HEYTIG, C., BLIEK, J., DE KRAKER, J., COAD, N., DE PAGTER-HOLTHUIZEN, P. & PEARSON, P.L. (1988). Molecular nature of genetic changes resulting in loss of heterozygosity of chromosome 11 in Wilms' tumours. *Hum. Genet.*, **81**, 41–48.

MANNENS, M., DEVILEE, P., BLIEK, J., MANJES, I., DE KRAKER, J., HEYTING, C., SLATER, R.M. & WESTERVELD, A. (1990). Loss of heterozygosity in Wilms' tumours, studied for six putative tumour suppressor regions, is limited to chromosome 11. *Cancer Res.*, **50**, 3279–3283.

MAW, M.A., GRUNDY, P.E., MILLOW, L.J., ECCLES, M.R., DUNN, R.S., SMITH, P.J., FEINBERG, A.P., LAW, D.J., PATERSON, M.C., TELZEROW, P.E., CALLEN, D.F., THOMPSON, A.D., RICHARD, R.I. & REEVE, A.E. (1992). A third Wilms' tumour locus on chromosome 16q. *Cancer Res.*, **52**, 3094–3098.

OGAWA, O., ECCLES, M.R., SZETO, J., MCNOE, L.A., YUN, K., MAW, M.A., SMITH, P.J. & REEVE, A.E. (1993). Relaxation of insulin-like growth factor II gene imprinting implicated in Wilms' tumour. *Nature*, **362**, 749–751.

ORKIN, S.H., GOLDMAN, D.S. & SALLAN, S.E. (1984). Development of homozygosity for chromosome 11p markers in Wilms' tumour. *Nature*, **309**, 172–174.

PELLETIER, J., BRUENING, W., LI, F.P., HABER, D.A., GLASER, T. & HOUSMAN, D.E. (1991). WT1 mutations contribute to abnormal genital system development and hereditary Wilms' tumour. *Nature*, **353**, 431–434.

PING, A.J., REEVE, A.E., LAW, D.J., YOUNG, M.R., BOEHNKE, M. & FEINBERG, A.P. (1989). Genetic linkage of Beckwith–Wiedemann syndrome to 11p15. *Am. J. Hum. Genet.*, **44**, 720–723.

RAINER, S., JOHNSON, L.A., DOBRY, C.J., PING, A.J., GRUNDY, P.E. & FEINBERG, A.P. (1993). Relaxation of imprinted genes in human cancer. *Nature*, **362**, 747–749.

REEVE, A.E., SIH, S.A., RAIZIS, A.M. & FEINBERG, A.P. (1989). Loss of allelic heterozygosity at a second locus on chromosome 11 in sporadic Wilms' tumour cells. *Mol. Cell Biol.*, **9**, 1799–1803.

RICCARDI, V.M., SUJANSKY, E., SMITH, A.C. & FRANCKE, U. (1978). Chromosome imbalance in the aniridia-Wilms' tumour association: 11p interstitial deletion. *Pediatrics*, **61**, 604–610.

RICCARDI, V.M., HITTNER, H.M., FRANCKE, U., YUNIS, J.J., LEDBETTER, D. & BURGESS, W. (1980). The aniridia-Wilms tumor association: the critical role of chromosome band 11p13. *Cancer Genet. Cytogenet.*, **2**, 131–137.

SAMBROOK, J., FRITSCH, E.F. & MANIATIS, T. (1989). *Molecular Cloning: A Laboratory Manual*, 2nd edn, Vol. 3. Cold Spring Harbor Laboratory Press: Cold Spring Harbor, NY.

SANTOS, A., OSORIO-ALMEIDA, L., BAIRD, P.N., SILVA, J.M., BOAVIDA, M.G. & COWELL, J.K. (1993). Insertional inactivation of the *WT1* gene in tumour cells from a patient with WAGR syndrome. *Hum. Genet.*, **92**, 83–86.

SCHWARTZ, C.E., HABER, D.A., STANTON, V.P., STRONG, L.C., SKOLNICK, M.H. & HOUSMAN, D.E. (1991). Familial predisposition to Wilms' tumour does not segregate with the WT1 gene. *Genomics*, **10**, 927–930.

SHEN, J.-C., RIDEOUT, W.M. & JONES, P.A. (1992). High frequency mutagenesis by a DNA methyltransferase. *Cell*, **71**, 1073–1080.

TADOKARO, K., FUJII, H., OHSHIMA, A., KAKIZAWA, Y., SHIMIZU, K., SAKAI, A., SUMIYOSHI, K., INOUE, T., HAYASHI, Y. & YAMADA, M. (1992). Intragenic homozygous deletion of the WT1 gene in Wilms' tumour. *Oncogene*, **7**, 1215–1221.

TON, C.C.T., HUFF, V., CALL, K.M., COHN, S., STRONG, L.C., HOUSMAN, D.E. & SAUNDERS, G.F. (1991). Smallest region of ovelap in Wilms' tumor deletions uniquely implicates an 11p13 zinc finger gene as the disease locus. *Genomics*, **10**, 293–297.

THOMPSON, A.D., SHEN, Y., HOLMAN, K., SUTHERLAND, G.R., CALLEN, D.F. & RICHARDS, R.I. (1992). Isolation and characterisation of $(AC)_n$ microsatellite genetic markers from human chromosome 16. *Genomics*, **13**, 402–408.

TURLEAU, C., DE GROUCHY, J., CHAVIN-COLIN, F., MARTELLI, H., VOYER, M. & CHARLAS, R. (1984). Trisomy 11p15 and Beckwith–Wiedemann syndrome: a report of two cases. *Hum. Genet.*, **67**, 219–221.

WADEY, R.B., PAL, N.P., BUCKLE, B., YEOMANS, E., PRITCHARD, J. & COWELL, J.K. (1990). Loss of heterozygosity in Wilms' tumour involves two distinct regions of chromosome 11. *Oncogene*, **5**, 901–907.

WAZIRI, M., PATIL, S.R., HANSON, J.W. & BARTLEY, J.A. (1983). Abnormalities of chromosome 11 in patients with features of Beckwith–Wiedemann syndrome. *J. Pediatr.*, **102** (6), 873–876.

WIEDEMANN, H.R. (1964). Complexe malformatif familial avec hernie ombilicle et macroglossie: un syndrome nouveau? *J. Genet. Hum.*, **13**, 223–232.